# Effectiveness of sodium hyaluronate eye gel in patients with dry eye disease: a multi-centre, open label, uncontrolled study

**DOI:** 10.12669/pjms.294.3487

**Published:** 2013

**Authors:** Nasir Saeed, ZA Qazi, Nadeem H Butt, Ahson Siddiqi, Neeta Maheshwary, Muhammad Athar Khan

**Affiliations:** 1 Dr. Prof. Nasir Saeed, Head Department of Ophthalmology, Hayat Abad Medical Complex Peshawar, Pakistan.; 2 Dr. ZA Qazi, LRBT Hospital, Township, Lahore, Pakistan.; 3 Dr. Nadeem H Butt, Department of Ophthalmology, Jinnah Hospital, Lahore, Pakistan.; 4 Dr. Ahson Siddiqi, Head Medical & Regulatory Affairs, 15 West wharf Road, Novartis Pharma Pakistan, Karachi, Pakistan.; 5 Dr. Neeta Maheshwary, Medical Scientific Liaison, 15 West wharf Road, Novartis Pharma Pakistan, Karachi, Pakistan.; 6 Dr. Muhammad Athar Khan, Lecturer, Department of Medical Education, King Saud bin Abdulaziz University, Riyadh, Kingdom of Saudi Arabia.

**Keywords:** Fluorescein eye stain, Schirmer's test, Tear Film Break up Time

## Abstract

***Objective:*** To assess the effectiveness of sodium hyaluronate Eye Gel in Dry Eye Disease (DED) patients

***Methods:*** A Multi-center, Open-label, Uncontrolled clinical trial was conducted in different centers of Pakistan. Ten ophthalmologists conducted this study in which 250 diagnosed patients with dry eye disease were enrolled after obtaining a written informed consent. Ten patients were dropped out during the study period. All patients were assessed as per following criteria for enrolling a patient into the study: Tear Film Break – up time (TBUT) ≤ 10 seconds, Schirmer Test – 1 ≤ 6 mm / 5 minutes and Positive Corneal Staining. Tolerability/safety was assessed by the monitoring and recording of all adverse events. The physical examination was done at baseline, 4^th^ week and 8^th^ week.

***Results*** ***:*** The mean age of the patients was 43.4 ±17.8 years and out of 240 patients 117 (48.7%) were males and 123 (51.3%) females. The Mean duration of symptoms was 19.3±23.9. At the initial visit the foreign body sensation was 50.6%, itching 35.9%, burning 50.6%, watering 42.9%, photophobia 25.3% and feeling of dryness in 14.7% of patients. After 4 weeks (2^nd^ visit), the symptoms were decreased to 47.1% foreign body sensation, 32.4% itching, 48.2% burning, 41.8% watering, 25.3% photophobia and 13.5% feeling of dryness. At the 3^rd^ visit (after 8 weeks) the frequency of symptoms were: 45.3% foreign body sensation, 30.6% itching, 45.9% burning, 40% watering, 24.7% photophobia and 13.5% feeling of dryness.

***Conclusion:*** Sodium Hyaluronate can provide a suitable alternate in the treatment of dry eye disease due to its reported efficacy on foreign body sensation, itching, burning, watering, photophobia and feeling of dryness,

## INTRODUCTION

The Dry eye disease (DED) is a common health problem which has an effect on the quality of life.^1^ The new definition of Dry Eye was developed in the International Dry Eye Workshop (DEWS) 2007 and is based on etiology, mechanism, and severity of the disease. The dry eye is a “multifactorial disease of the tears and ocular surface that results in symptoms of discomfort, visual disturbance, and tear film instability with potential damage to the ocular surface. It is accompanied by increased osmolarity of the tear film and inflammation of the ocular surface”.^2^

Dry eye is a common ocular problem and also an important public health problem. DED patients are having problems in daily routine activities such as reading, working on a computer, and car driving. DED also increases the risk of ocular infections, ocular discomfort, fatigue, and visual disturbance.^3^^, ^^4^ Only a few population based studies are available regarding the magnitude of the problem. The studies conducted in US to determine the prevalence of DED showed 14.6% (Salisbury Eye Study).^5^ The reported frequency of dry eye syndrome was found 16%.in adult patients attending the eye clinic of tertiary care hospital in Pakistan.^6^

The important risk factors for DED are older age, female sex, reduced androgen levels, exogenous estrogen use, and an imbalance in the dietary intake of essential fatty acids as identified by the studies over the past decade.^7^^-^^8^ A study conducted on elderly Korean population found that female sex, age, and hormonal influence were risk factors for both DED and depression.^9^ Wang et al and Galor et al, reported a relationship between depression and DED.^10^^,^^11^

Hyaluronan (HA) has distinctive viscoelastic and hygroscopic properties and plays many important roles in the human body. It is a naturally occurring lubricant and is made up of biocompatible polysaccharide It is because of its excellent water-retaining properties that it is used in ophthalmic products.^12^^-^^14^ HA is found mainly in the connective tissue of the skin, inside the umbilical cord, synovial fluid in joints and eye. In eye, it is found in the vitreous, lacrimal gland, cornea, conjunctiva, and tear fluid. HA has an anti-inflammatory and wound healing property. HA inhibits free radicals thus prevents oxidative damage to cells.^15^^,^^16^

The objective of this study was to assess the effectiveness of sodium hyaluronate Eye Gel in diagnosed patients of DED.

## METHODS

A Multi-center, Open-label, Uncontrolled clinical trial was conducted in different centers of Pakistan. Ten ophthalmologists conducted this study in which 250 diagnosed patients with dry eye disease were included after obtaining a written informed consent. All patients were assessed as per following criteria for enrolling a patient into the study: Presence of at least 2 of the 6 symptoms (Foreign Body Sensation, Itching, Burning, Watering, Photophobia and Feeling of Dryness in the eye) and at least one of the 3 tests, Tear Film Break – up time (TBUT) ≤ 10 seconds, 2) Schirmer Test – 1 ≤ 6 mm / 5 min and Positive Corneal Staining. Patients with Entropion, Ectropion, Trichiasis, or using Cortico-steroids drugs were excluded from the study.

Sodium hyaluronate Eye Gel 2-4 times a day was prescribed to patients for 8 weeks. Tolerability/safety was assessed by the monitoring and recording of all adverse events. The physical examination was performed at baseline, 4^th^ week and 8^th^ week. The confidentiality of data and patient anonymity was protected by the codes given in the study documents.

Demographic and baseline variables were described by mean + standard deviation for continuous data and frequency and percentages were calculated for categorical data. One Way ANOVA was performed and p value <0.05 was considered as significant.

## RESULTS

Ten patients dropped out during the study period from a total of 250 diagnosed patients of Dry Eye and the response rate was 96%. The mean age of the patients was 43.4 ±17.8 years. Out of 240 patients, 117 (48.7%) were males and 123 (51.3%) females. The Mean duration of symptoms was 19.3 ±23.9 months. At the baseline, foreign body sensation 50.6%, itching 35.9%, burning 50.6%, watering 42.9%, photophobia 25.4% and feeling of dryness 14.7%.

After 4 weeks (2^nd^ visit) the results showed that the foreign body sensation 47.1%, itching 32.4%, burning 48.2%, watering 41.8%, photophobia 25.3% and feeling of dryness 13.5%. After 8 weeks (3^rd^ visit) the changes in ocular symptoms were: foreign body sensation 45.3%, itching 30.6%, burning 45.9%, watering 40%, photophobia 24.7% and feeling of dryness 13.5%. The Mean TBUT was 6.6±2.2 in the first visit, 8.3±2.2 second visit and 10.1±2.3 seconds in the third visit (p-value <0.05). Schirmer’s test was 9.1 ±5.7 in the first visit, 10.6 ±5.3 in the second visit and 11.8±4.1 in the third visit (p-value <0.05).The above results showed a significant difference in the ocular symptoms between the visits.

**Table-I:** Change in Ocular symptoms and signs with visit (N = 240

*Ocular History*	*Visit-1 (Baseline)*		*Visit-2 (Week 4)*		*Visit-3 (Week 8)*	
	*N*	*%*	*N*	*%*	*N*	*%*
Foreign Body sensation	121	50.6%	113	47.1%	109	45.3%
Itching	86	35.9%	78	32.4%	73	30.6%
Burning	121	50.6%	116	48.2%	110	45.9%
Watering	103	42.9%	100	41.8%	96	40.0%
Photophobia	61	25.4%	61	25.3%	59	24.7%
Feeling of Dryness	35	14.7%	32	13.5%	32	13.5%

**Table-II:** Change in Tear film breakup time and Schirmer's Test with visits (N = 240

	*Tear film breakup time (sec)*			*Schirmer's Test mm/5 min (Without Anesthesia)*		
	*Visit-1 (Baseline)*	*Visit-2 (Week 4)*	*Visit-3 (Week 8)*	*Visit-1 (Baseline)*	*Visit-2 (Week 4)*	*Visit-3 (Week 8)*
N	216	217	212	207	212	208
Mean	6.6	8.3	10.1	9.1	10.6	11.8
Median	7.0	9.0	10.0	8.0	5.3	12.0
Std. Deviation	2.2	2.2	2.3	5.7	3.5	4.1
p-value	<0.05			<0.05		

## DISCUSSION

The results of this study showed that sodium hyaluronate-containing eye gel reduces the ocular symptoms in patients with dry eye. Treatment with 0.4% hyaluronic acid hypotonic solution drops gave better results in relieving symptoms, along with a statistically significant improvement (p < 0.001) in the state of the corneo-conjunctival epithelium, than the isotonic solution.^16^ Pasquale et al found that sodium hyaluronate significantly improved signs and symptoms in patients with moderate to severe dry eye independently from the saline composition of the ophthalmic solutions.^17^

In vitro hyaluronate promotes cell migration and can stabilize ocular surface epithelial barrier suggesting that it may be directly involved in the process of epithelial repair by activation of the CD44 (the hyaluronate receptor).^18^^,^^19^ Brignole and associates found that comfort was better in Sodium hyaluronate (SH) group as compared to carboxymethylcellulose (CMC) group. SH group also showed faster recovery in keratitis (type, extent and depth) and symptoms than in the CMC group.^20^ A comparative study between diquafosol group and sodium hyaluronate group also showed that the SH group had lower adverse reactions such as eye discharge and eye irritation than in the other group.^21^

Johnson and coworkers compared the effect of saline and 0.1% and 0.3% HA on TBUT. A significant improvement was found in TBUT up to 6 hours. They also noticed that the 0.3% HA had greater effect on TBUT as well as on symptoms (P < 0.04).^22^ Our study also showed the improvement of mean TBUT from 6.6 second (SD±2.2) in the first visit to 10.1 seconds (SD±2.3) in the third visit (p-value <0.05). Prabhasawat and coworkers also found the increase in TBUT in HA group at 30 and 60 minutes (P < 0.04 and P < 0.005, respectively).^23^ Our study found that the foreign body sensation, itching, burning, watering, photophobia, and feeling of dryness decreased after 8 weeks of treatment with HA. Dumbleton and associates also reported the efficacy of HA with a significant difference in subjective symptoms in the group using HA.^24^

Literature review showed that HA is found throughout the body and can be used to mimic the natural tears in such products as artificial tears. Previous studies also show that HA-based ophthalmic preparations can serve as natural tears.^25^

## CONCLUSION

In conclusion, this study suggests that sodium hyaluronate Eye Gel has a beneficial effect on the foreign body sensation, itching, burning, watering, photophobia and feeling of dryness in a well defined and homogeneous population of patients with dry eye, selected by using stringent inclusion criteria. No adverse event with sodium hyaluronate eye gel was reported by the patients.

## Authors Contribution:

Prof. Dr. Nasir Saeed: Design, drafting, data collection, critical review.

Dr. ZA Qazi: Data collection, writing the results, drafting.

Dr. Nadeem H Butt: Writing the discussion, Drafting, Data collection.

Dr. Ahson Siddiqi: Design, critical review.

Dr. Neeta Maheshwary: Data collection, Data analysis.

Dr. Muhammad Athar Khan: Data analysis, writing the results.

## Notes

***Limitations of the Study: ***The limitations of this study were lack of control group and also the open label nature of the study. The equal number of male and female patients will have an effect on the results because some of the factors e.g. gender and menopause is associated with female gender. The duration (8 weeks) of the study was also a limitation to observe the long-term effects of the treatment.
